# Relationship between delayed luminescence emission and mitochondrial status in *Saccharomyces cerevisiae*

**DOI:** 10.1038/s41598-021-04290-9

**Published:** 2022-01-10

**Authors:** Miao Tian, Qing Li, Yang Liu, Peng Zheng, Danyu Li, Yanpeng Zhao, Bing Wang, Chenhao Li, Jing Wang, Peng Gao, Qing Tang, Xiaochun Zhang, Hong Wu

**Affiliations:** ENNOVA Institute of Life Science and Technology, ENN Group, South District of ENN Industrial Park, Langfang, 065001 Hebei China

**Keywords:** Biochemistry, Biophysics, Microbiology

## Abstract

Delayed luminescence (DL) is gradually used in various detection of biological systems as a rapid detection technique, however, its biological mechanism was still not clear. In this study, a new model of DL detection system for liquid biological samples is established to investigate the DL emission of *Saccharomyces cerevisiae* cells cultured in different glucose concentrations. We analyzed the relationship between the DL emission and cell growth, cell vitality, mitochondrial morphology, mitochondrial DNA (mtDNA) copy number, adenosine triphosphate (ATP), oxygen consumption rate (OCR), as well as mitochondria membrane potential (MMP) in *S. cerevisiae* cells cultured with 0.01, 0.05, 0.15, 3, 10 and 20 g/L glucose respectively. It was found that the DL emission had strong correlation with mitochondrial morphology, OCR, and MMP. The results suggested that DL is an indicator of mitochondria status under different glucose supply conditions, and may be an effective method to detect mitochondrial metabolism related disorders.

## Introduction

Ultraweak biophoton emission is a common phenomenon of life, which exists in all kinds of animals, plants and microorganisms^1–7^. Emission of biophoton is highly sensitive to the changes in the internal biological system and the influence of the external environment, so through the detection and analysis of biological photons, the internal microscopic information inside organisms can be obtained. Delayed luminescence (DL), a detection method of ultra-weak luminescence in biological systems, is known as the long-lasting afterglow (usually on a time scale of microseconds to seconds) of biological systems that are illuminated by white or monochromatic light. The DL detection has better signal and sensitivity than spontaneous photon emission (SPE), make it an ideal choice for a reliable and inexpensive optical biopsy technique. The application of DL detection technologies had been well studied in many fields, including food safety inspection^8^, natural organic matter detection^9^, water pollution detection^10^, physiological characteristics of plants detection^11^, and seed quality measurement and analysis^12,13^, Chinese herbal medicine identification^14,15^, clinical application, human health^16–18^ and so on. At the same time, some theoretical progress has been made based on these technical application. Some studies suggested that DL was directly generated from autofluorescence emitters such as flavins^19^, from solitons in hierarchically organized structures such as the cytoskeleton^4^, or from collective molecular interactions such as in mitochondrial membrane protein complexes^20^. Relationship between DL and mitochondria was reported in several papers. According to Kim report^21^, DL originated from mitochondrial complex III under NIR-induced condition and from mitochondrial complex I under UV–Vis induced condition. Baran et al.^22^ used mitochondrial complex I targeting agent rotenone, menadione, and quercetin to probe the DL of human leukemia Jurkat T cells. The authors suggested that DL of leukemia Jurkat T cells originated mainly from mitochondrial complex I. Researches^18,20,23,24^ on DL in cancer showed that cancer cells had higher levels of DL emission than normal cells. Further theoretical analysis indicated that DL was closely related to cell apoptosis and oxidative stress. In the mechanism analysis, it is considered that NADH, FMN, Fe/S centers and Ubiquinone in mitochondria are the influencing factors of DL. It can be seen from the above review that the research interest in mitochondria and DL has increased in recent years and some progress has been made, but the precise biological mechanism remains to be further studied.

*Saccharomyces cerevisiae* (*S. cerevisiae*) is a model organism and has been widely used in theoretical research. The metabolism of *S. cerevisiae* was originally called “aerobic glycolysis”, also known as the “Warburg effect”^25^. However, it was later discovered that the Crabtree effect^26^ also exists in the metabolic pathway of *S. cerevisiae* cells. The Crabtree effect is characterized by an increase of glycolytic flux, repression of mitochondrial respiration, and establishment of fermentation as a major pathway for ATP production in the presence of high concentrations of fermentable carbohydrates^27^. In contrast, under low concentrations of fermentable carbohydrates, *S. cerevisiae* cells activate tricarboxylic acid (TCA) cycle, electron transfer chain (ETC), and oxidative phosphorylation (OXPHOS) genes and switch metabolism from fermentation to respiration^28,29^. Mitochondria are the main sites of cellular oxidative respiration, therefore the morphology, quantity and activity of mitochondria are directly affected by different fermentable carbohydrates concentrations. Because of this feature, *S. cerevisiae* is a good candidate to study the relationship between mitochondrial status and DL.

In this study, a DL detection system that suitable for liquid biological sample was established and for the first time, to our knowledge, the correlation between DL emission and the glucose-induced mitochondrial characteristics was studied.

## Results

### DL emission

**Figure 1:**
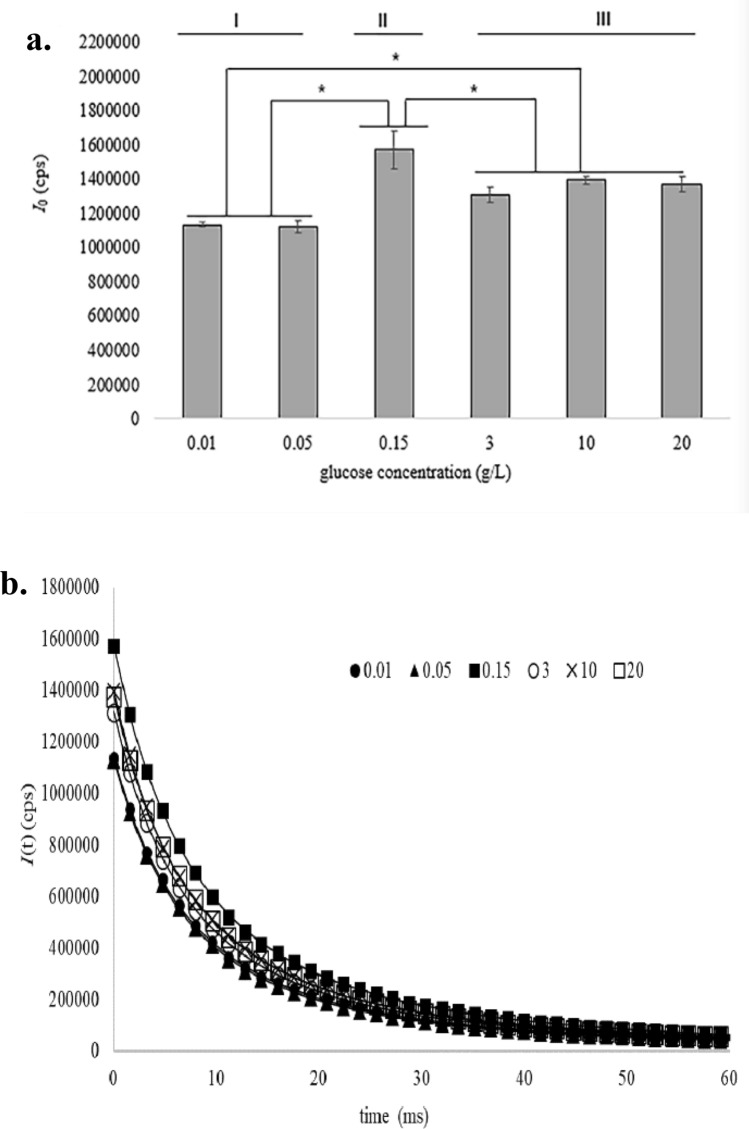
*I*_0_ (**a**) and kinetics curves (**b**) of DL emission from *S. cerevisiae* cells under 0.01, 0.05, 0.15, 3, 10 and 20 g/L glucose for 3 h. Statistical analyses were performed using one-way ANOVA followed by Tukey’s test. *p < 0.05 indicated statistically significant differences of *I*_0_ among the yeast cells cultured in different glucose concentrations. Values represent the mean ± SD of three independent experiments. Group categorization was performed by K-means clustering, group I included cells in 0.01 and 0.05 g/L glucose, group II included cells in 0.15 g/L glucose, group III include cells in 3, 10 and 20 g/L glucose.

The DL emitted from yeast cells which cultured with 0.01, 0.05, 0.15, 3, 10 and 20 g/L glucose respectively for 3 h was recorded after 405 nm laser excitation and evaluated in ENN software. The result was showed in Fig. 1. It appeared that glucose concentration significantly affected the DL emission of yeast cells. According to DL initial intensity *I*_0_ (Fig. 1a), the cells could be classified into three groups. The group I was cells cultured with 0.01 and 0.05 g/L glucose, the cells in this group have the lowest *I*_0_. The group II was cells with 0.15 g/L glucose. The cells in this group have the highest *I*_0_, which was nearly 1.4 times higher than that of group I and 1.1 times higher than that of group III. The group III was cells cultured with 3, 10 and 20 g/L glucose, and the *I*_0_ of cells in this group was in the middle compared with that in other groups. Although the dynamic curves (Fig. 1b) of cells cultured with different concentration of glucose tended to be the same after 30 ms, their dynamic curves were different within the initial 30 ms. According to the kinetic curves, the cells were also classified visually into three groups, which corresponded perfectly to the groups based on *I*_0_. Cells cultured with 0.01 and 0.05 g/L glucose were in one group, and there was no significant difference in the relaxation trend of DL kinetic curves between the two kinds of cells in this group. Cells cultured with 3, 10 and 20 g/L glucose were in another group, and there was no significant difference in the relaxation trend of DL kinetic curves among the three kinds of cells in this group. Cells cultured with 0.15 g/L glucose were in one group alone.

### Cell growth and cell vitality

**Figure 2:**
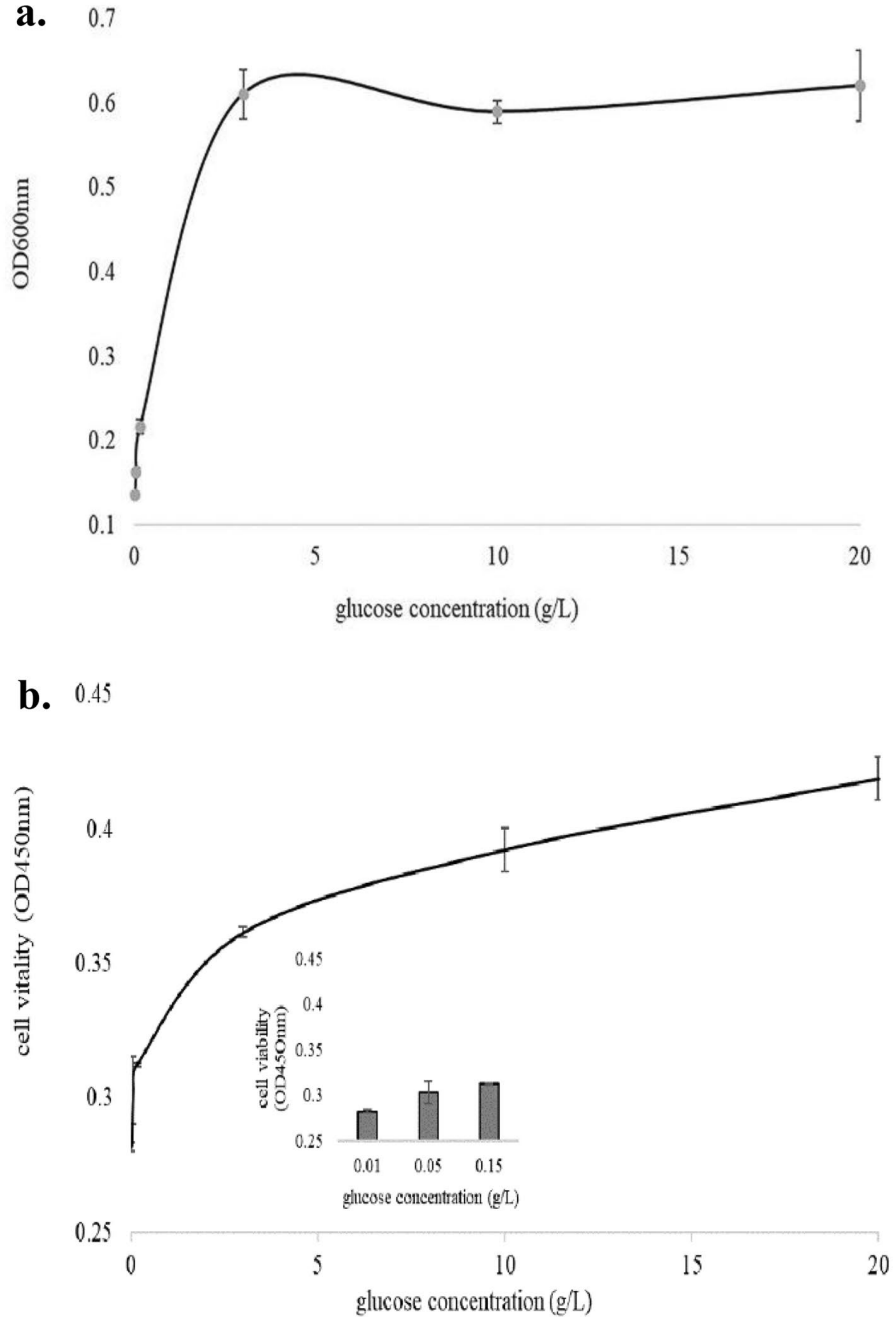
Cell growth (**a**) and cell vitality (**b**) of the yeast cells cultured in 0.01, 0.05, 0.15, 3, 10 and 20 g/L glucose for 3 h. Inserted data in Fig. 2b represented cell vitality of yeast cells cultured in 0.01, 0.05, and 0.15 g/L glucose for 3 h respectively.

To confirm the effect of different glucose concentration on bioenergy and biochemical reaction, we detected the growth and vitality of yeast cells cultured in different glucose concentrations. The result (Fig. 2a) showed that in low concentration of glucose (0.01, 0.05 and 0.15 g/L), the cell growth was slow, but the cell growth increased as the concentration of glucose increased. When concentration of glucose was 3 g/L, the cell growth was at the highest level. In addition, cell growth was the same under 3, 10 and 20 g/L of glucose conditions, indicating that 3 g/L of glucose was sufficient for cell growth and that glucose was no longer a limiting factor for cell growth. And we measured the residual glucose content in the culture medium after the yeast cells were cultured at different glucose concentrations for 3 h. We found that the glucose in the medium was not completely consumed, the residual glucose concentrations in the medium were 0.008, 0.02, 0.053, 1.866, 8.671 and 17.052 g/L under 0.01, 0.05, 0.15, 3, 10 and 20 g/L glucose respectively, directly indicating that glucose was no longer a limiting factor for cell growth. Figure 2b showed that cell vitality increased as the concentration of glucose increased. The cell activity here reflected the level of energy metabolism of cells. High cell activity meant higher cell growth potential. The trend of cell activity corresponded well with the trend of cell growth (r = 0.94).

### Mitochondrial DNA (mtDNA) copy number and morphology imaging

**Figure 3:**
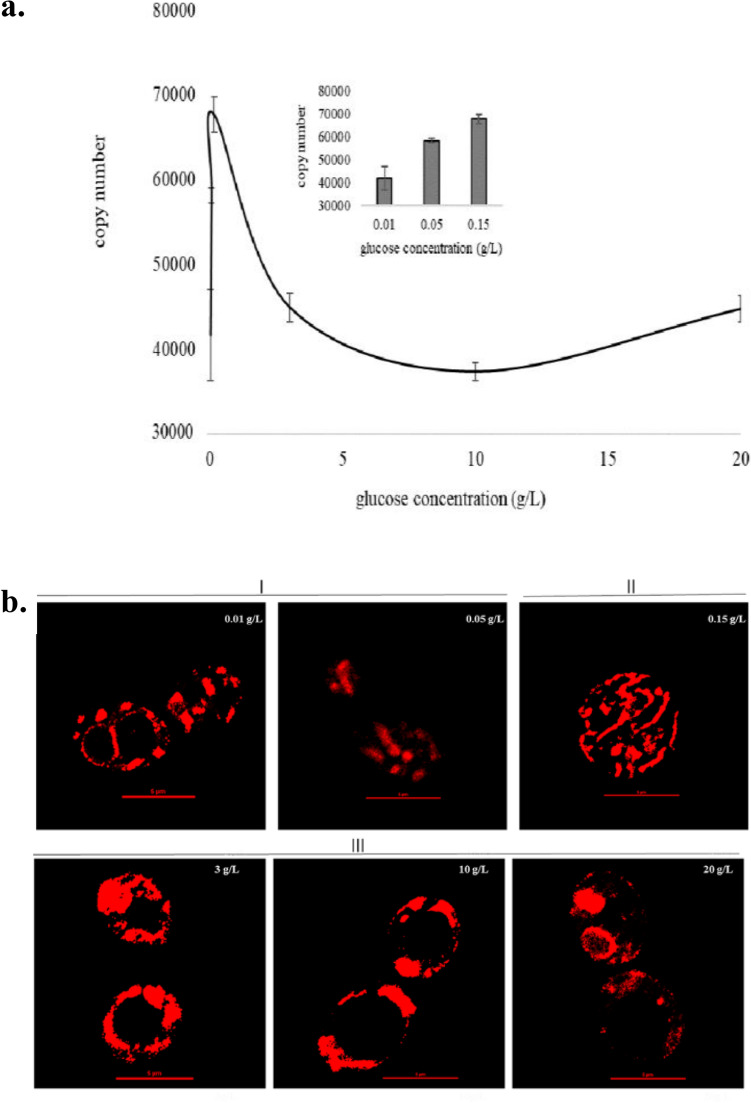
Mitochondrial DNA (mtDNA) copy number (**a**) and morphology imaging (**b**) of yeast cell cultured in 0.01, 0.05, 0.15, 3, 10 and 20 g/L glucose for 3 h. Insert data in Fig. 3a represented mtDNA copy number of yeast cells in 0.01, 0.05, and 0.15 g/L glucose for 3 h. Mitochondrial morphology in (**b**) were divided into three groups, group I included cells under 0.01 and 0.05 g/L glucose, group II included cells under 0.15 g/L glucose, group III included cells under 3, 10 and 20 g/L glucose. Scale bar, 5 μm.

To elucidate the relationship between mitochondria and DL emission, we detected mtDNA copy number and morphology changes in different concentration of glucose. MtDNA copy number firstly increased and then decreased with the increase of glucose concentration (Fig. 3a). When the glucose concentration was 0.15 g/L, the mtDNA copy number was the highest. The mitochondria of yeast cells were stained with fluorescent dye and observed by confocal microscope. We conducted three repeated experiments with three replicates for each experiment, and repeated samples were taken for each sample during mitochondrial morphology observation. Therefore, there were dozens of mitochondrial samples observed under each glucose concentration. Visually, 90% of mitochondrial morphology was shown in Fig. 3b. Visually, mitochondrial morphology in Fig. 3b were divided into three groups. Group I included cells under 0.01 and 0.05 g/L glucose and the mitochondria morphology in this group presented multi-point dispersion. Group II included cells under in 0.15 g/L glucose and the mitochondrial showed a uniformly distributed network structure. The mitochondria in group III (3, 10 and 20 g/L) tend to cluster together and form one or more large structures.

### Adenosine triphosphate (ATP) detection

**Figure 4:**
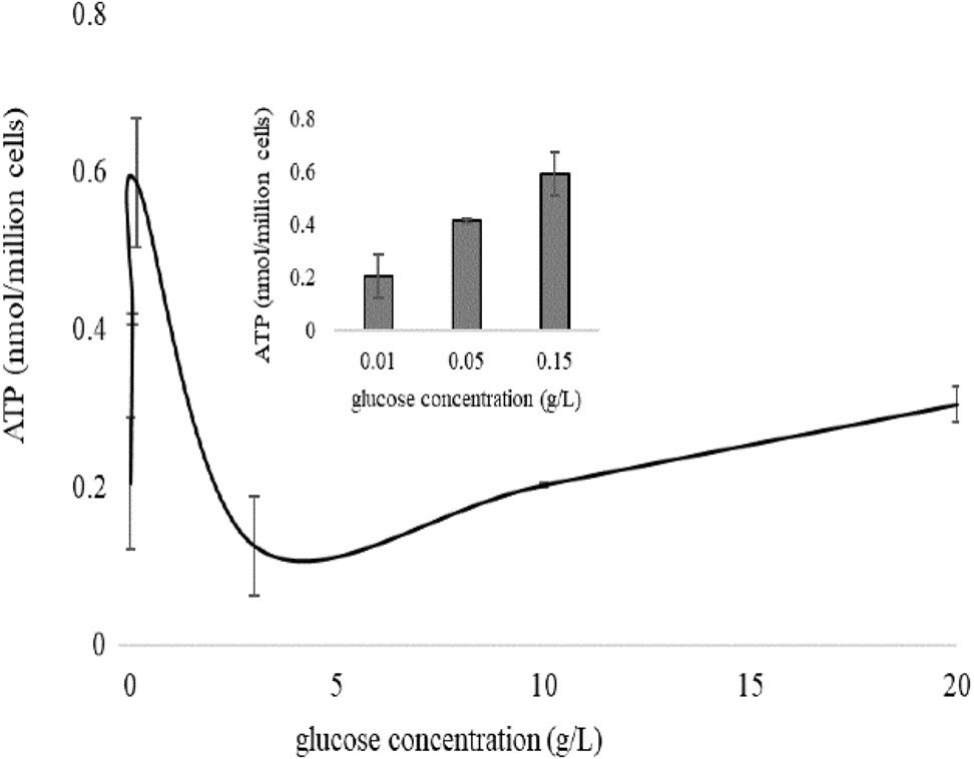
Adenosine triphosphate (ATP) content of yeast cells cultured in 0.01, 0.05, 0.15, 3, 10 and 20 g/L glucose for 3 h. Insert data represented ATP content of yeast cells in 0.01, 0.05, and 0.15 g/L glucose for 3 h.

ATP is the direct energy molecule to maintain cell growth. To investigate the content of ATP under different glucose concentrations, total cell extraction was analyzed through high performance liquid chromatography (HPLC). The intercellular ATP increased with the increase of glucose concentration under 0.01–0.15 g/L glucose concentration. The content of ATP was the highest when the glucose concentration was 0.15 g/L, and then decreased as the glucose concentration increased to 3 g/L. However, the content of ATP showed a slow upward trend when the glucose concentration was higher than 3 g/L (Fig. 4). When glucose concentration was low, yeast cells were predominantly respiring and produce high amounts of ATP, but when glucose concentration was high, here above 3 g/L, yeast cells were predominantly glycolytic and produce less ATP than they were respiring. In glycolysis dominated metabolic mode, cell vitality risen with increasing glucose concentration, while cell growth did not differ significantly (Fig. 2). In addition, the content of ethanol increased with the increase of glucose concentration (data not shown), suggesting the fermentation capacity of cells corresponded well with the glucose concentration. As an indicator of cell global metabolic viability, ATP reflects the metabolic status of cells, including cell activity, cell growth, fermentation capacity and so on.

### Oxygen consumption rate (OCR) detection

**Figure 5:**
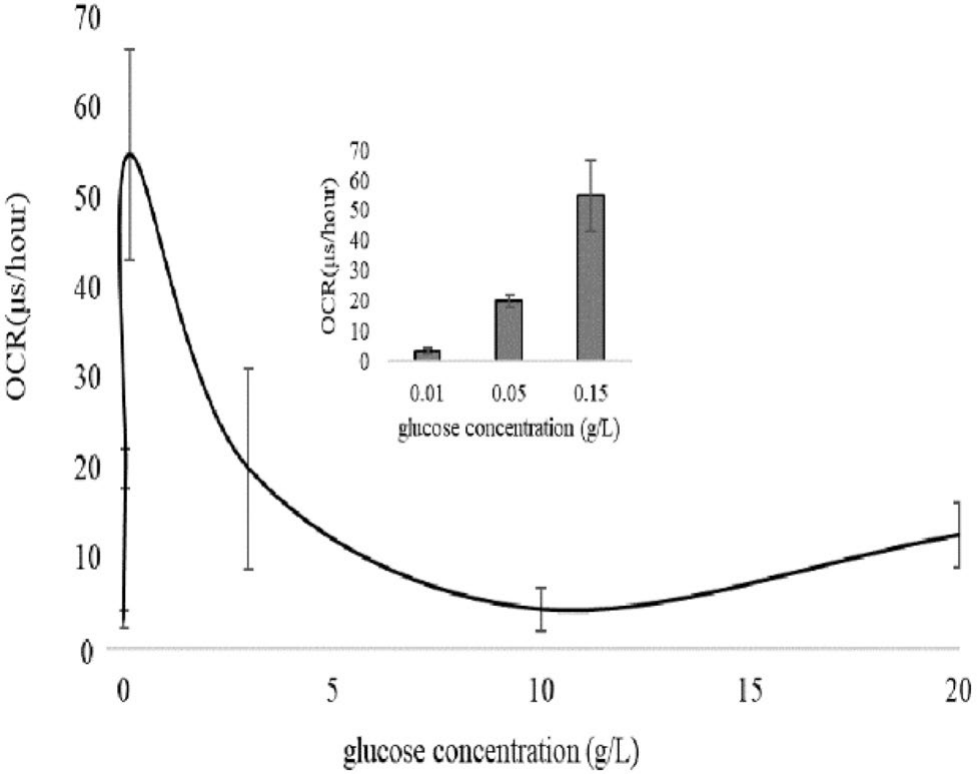
Oxygen consumption rate (OCR) of yeast cells cultured in 0.01, 0.05, 0.15, 3, 10 and 20 g/L glucose for 3 h. Insert data represents OCR of yeast cells under 0.01, 0.05, and 0.15 g/L glucose for 3 h.

Oxygen consumption is an indicator of the function of the OXPHOS activity. In order to evaluate whether the concentration of glucose is related to DL emission, OCR was performed in yeast cells with the different levels of glucose. The phosphorescent oxygen-sensitive probe, MitoXpress, was used to detect OCR. The result was expressed as a slope and the unit was μs/h. The result (Fig. 5) showed that OCR increased with the glucose concentration from 0.01 to 0.15 g/L and then decreased with the glucose concentration from 3 to 20 g/L. OCR was highest when the concentration of glucose was 0.15 g/L.

### Mitochondrial membrane potential (MMP) detection

**Figure 6:**
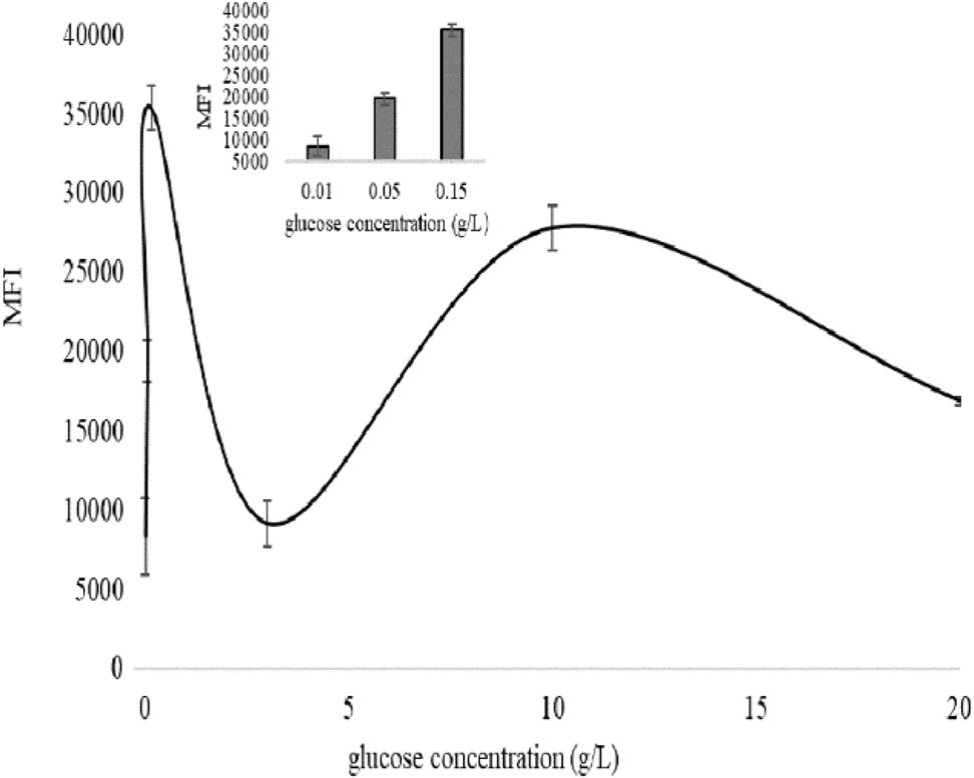
Mitochondrial membrane potential (MMP) of yeast cells cultured in 0.01, 0.05, 0.15, 3, 10 and 20 g/L glucose for 3 h. Insert data represented MMP of yeast cells in 0.01, 0.05, and 0.15 g/L glucose for 3 h.

MMP is an another important indicater of mitochondria activity. So, in this work, fluorescent probe Rhodamine123 (Rho123) was used to detect MMP at different glucose concentrations. Mean fluorescence intensity (MFI) showed a trend of first increasing and then decreasing, then increasing and then decreasing again with the increase of glucose concentration. Under low concentration of glucose (0.01–0.15 g/L), the MFI of yeast cells under 0.15 g/L glucose concentration was the highest. While, under high concentration of glucose (3–20 g/L), the highest MFI was found in yeast cells in 10 g/L (Fig. 6).

### Correlation analysis

Table 1 presents the correlations among all variables included in this study. The correlation coefficient (r) between OCR and *I*_0_ was 0.65, and between MMP and *I*_0_ was 0.74, indicating that *I*_0_ directly correlated with OCR and MMP. OCR was significantly related to mtDNA copy number (r = 0.91) and ATP (r = 0.82). Cell growth and cell vitality had the highest correlation (r = 0.94). The correlation coefficient of ATP and MMP was 0.73. The correlation coefficient between OD600 or cell viability and mitochondrial parameters was negative or very low, showing no statistically significant.

**Table 1:** Correlation analyses. Pearson correlation was analyzed by SPSS25.0 software.

	*I*_0_	MtDNA copy number	Cell growth	OCR	Cell vitality	MMP	ATP
*I*_0_	1						
MtDNA copy number	0.34	1					
Cell growth	0.35	− 0.56	1				
OCR	0.65*	0.91**	− 0.30	1			
Cell vitality	0.41	− 0.47	0.94***	− 0.26	1		
MMP	0.74*	0.55	− 0.09	0.63	0.08	1	
ATP	0.44	0.92***	− 0.53	0.82**	− 0.33	0.73*	1

*p < 0.05.

**p < 0.01.

***p < 0.001.

## Discussion and conclusion

Glucose is the energy source of living cells and the main energy supply substance of living organisms. In *S. cerevisiae* species, cell growth and cell vitality of yeast cells was very sensitive to glucose concentration (Fig. 1), which was consistent with previously report^30^. It had shown that metabolism of yeast cells changed in response to different concentrations of glucose^31,32^, allowing the investigation of mitochondria changes under different energy supply conditions. Combined with the previous experiments, we investigated the changes in mitochondria and DL of yeast cells cultured in SC medium containing 0.01, 0.05, 0.15 3, 10 and 20 g/L glucose, respectively.

Under aerobic and high concentrations of glucose, *S. cerevisiae* undergoes fermentation. In contrast, under low concentrations of glucose, *S. cerevisiae* metabolizes glucose by mitochondria respiration^33^. In this study, Yeast cells metabolizes glucose predominantly by mitochondria respiration when they were cultured with 0.01, 0.05, or 0.15 g/L glucose. However, the DL initial intensity *I*_0_ of 0.01 and 0.05 g/L glucose group was significantly lower than that of 0.15 g/L glucose group. This may be because glucose levels are too low to provide enough energy for cells, resulting in low cellular respiration and DL emission. When glucose concentration was higher than 3 g/L, the yeast cells metabolized glucose mainly through glycolysis and fermentation. The DL emission of yeast cells in higher than 3 g/L glucose was lower than that of cells in 0.15 g/L glucose, but higher than that of 0.01 and 0.05 g/L glucose, and the differences of DL emission between cells undergoing vigorous respiration and cells undergoing fermentation were statistically significant.

In addition, under 0.15 g/L glucose conditions, the yeast cells had the largest mtDNA copy number, the highest ATP content, the highest OCR, and the highest MFI (MMP). The results indicated that mitochondria respiration of the cells was the strongest at 0.15 g/L glucose concentration. When glucose concentration was lower than 0.15 g/L or higher than 3 g/L, mitochondria respiration was weak in yeast cells, and the mtDNA copy number, ATP content, OCR, and MFI were also lower. Correlation analysis of all variables was performed. *I*_0_ was correlated with OCR and MMP, and OCR is, in turn, correlated with mtDNA copy number, suggesting the possibility that *I*_0_ can indirectly reflect mtDNA copy number. However, regardless of this, OCR, MMP, and mtDNA copy number are all important indicators of mitochondrial activity, so *I*_0_ is inextricably linked to intracellular mitochondria. We know cell growth and cell vitality can reflect the overall activity of cell, so we analyzed the relationship between cell growth or cell vitality and OCR, MMP and mtDNA copy number, but negative was found. We suggested that DL may tend to reflect local information inside the cells rather than the global metabolic activity of the cells under the conditions of this research.

Previous study^34^ showed that when growing in presence of glucose, mitochondria possessed a single mitochondrial network with extensive branching, once glucose was removed from the medium, mitochondria exhibited a dotted localization, like a series of globules connected by small bridges. In this study, the morphology of mitochondria under different glucose supply was analyzed in detail, and the results showed that the morphology of mitochondria was visually divided into three groups according to glucose concentration. The group I included mitochondria in 0.01 and 0.05 g/L glucose, the mitochondria in this group possess a dotted localization. The group II was mitochondria in 0.15 g/L glucose, the mitochondria exhibited an interwoven network structure. The rest were group III, and the mitochondria possessed a single mitochondrial network with extensive branching as described in the above literature^34^. In addition, as it was known that in the high concentration of glucose, yeast cells mainly consume glucose through glycolysis and fermentation, and mitochondria respiration is relatively inactive, mitochondrial morphology was a huge single mitochondrial network structure. At low concentrations of glucose, carbon flux enters mitochondria, and activates the Krebs cycle and OXPHOS. Mitochondrial respiration was very active, and mitochondrial morphology was an interwoven mitochondrial network structure. The grouping based on mitochondrial morphology was consistent well with that of DL, indicating that DL was highly correlated with mitochondrial morphology. Through comprehensive analysis of DL, biochemical parameters and mitochondrial morphology, our results further support the view that mitochondria are source of DL and DL can reflect cellular mitochondria metabolism under certain conditions.

As we know, ultraweak biophoton emission was first discovered in plants, where chloroplast was carrier of electron transport chains. In Joliot’ research^35^ on DL mechanism in *Chlorella pyrenoidosa*., they thought that DL is due to charge recombination between the oxidized donor and the reduced acceptor of PSII centers and they found both proton gradient and membrane potential in PSIII can affect the intensity of DL. Mitochondria are electron carriers in most eukaryotic cells, so we hypothesized that mitochondrial and DL must also be related. Grasso et al.^36^, Baran et al.^3,20,22^ and Kim et al.^21^ had performed some studies on the mechanism of DL. The authors treated the corresponding cells with exogenous intervention or drugs, and then measured some biochemical parameters and DL. It turns out that DL is related to mitochondria membrane protein complexes. Different from these studies, our study took advantage of the cheapness of metabolic transformation in yeast cells and studied the relationship between DL and mitochondria from the perspective of energy regulation, and finally found that DL could directly reflect mitochondrial morphology, OCR and MMP.

In conclusion, we established a new model of DL detection system and detected the changes of DL in *S. cerevisiae* under differernt glucose conditions, and also detected cell growth, cell vitality, mitochondrial morphology, mtDNA copy number, ATP content, OCR and MMP, and we carried out correlation analysis of theses indexes. All the results illustrated that DL was highly correlated with mitochondrial morphology, OCR and MMP, suggesting that DL is an effective method to detect mitochondrial function under different energy supply conditions. As the research gose further, DL may be developed as a means to dectect mitochondrial metabolic disorders and a rapid, non-invasive medical detection technique.

## Materials and methods

### Cell culture

The industrial instant dry yeast *S. cerevisiae* (Angel Yeast CO. Ltd., China) was used in this study. This commercial yeast strain has been isolated and purified by multi-generation monoclonal cloning. It was a fast growing, vigorous and stable strain with a doubling time of 1–1.5 h at 2% glucose concentration. The yeast cells were preserved in 15% glycerol at − 70 °C. Before each experiment, the yeast strain preserved in − 70 °C was inoculated into a 200 mL glass Erlenmeyer flasks containing 50 mL SC medium and incubated overnight at 30 °C and 150 rpm. The following day, the yeast cells were re inoculated into a new 200 mL glass Erlenmeyer flask containing 50 mL SC medium with a calculated final concentration of 0.0005 OD600nm (Optical density at 600 nm) and continued to be cultured overnight at 30 °C and 150 rpm. According to the growth curve, the yeast cells in logarithmic phase were obtained, which could be used in the subsequent experiments. During this period, we measured the glucose and alcohol contents of the cultures, and determined that logarithmic cells were harvested at first growth phase (fermentative metabolism). The formula of SC medium is as follows: Yeast Nitrogen Base Without Amino Acids (Product Number: Y0606) 6.7 g, Yeast Synthetic Drop-out Medium Supplements (Product Number: Y1501) 1.92 g, Uracil (Product Number: U1128) 0.076 g and D-(+)-glucose (Product Number: 47829) 20 g in a final volume of 1 L. All reagents were purchased from Sigma-Aldrich (Steinheim, Germany). All glassware and medium were autoclaved at 121 °C for 20 min.

After centrifugation, logarithmic grown cells were washed twice with SC medium without D-(+)-glucose and then inoculated into a 200 mL glass Erlenmeyer flask containing 50 ml SC medium at a final concentration of 0.1 OD600nm. The components of SC medium were the same as pre-culture medium except for the different concentrations of D-(+)-glucose (0.01 g/L, 0.05 g/L, 0.15 g/L, 3 g/L, 10 g/L, and 20 g/L respectively). The cells were then cultured at 30 °C and 150 rpm for 3 h. Three replicates were used for each experiment, and three independent experiments were performed.

### Experimental system design

**Figure 7:**
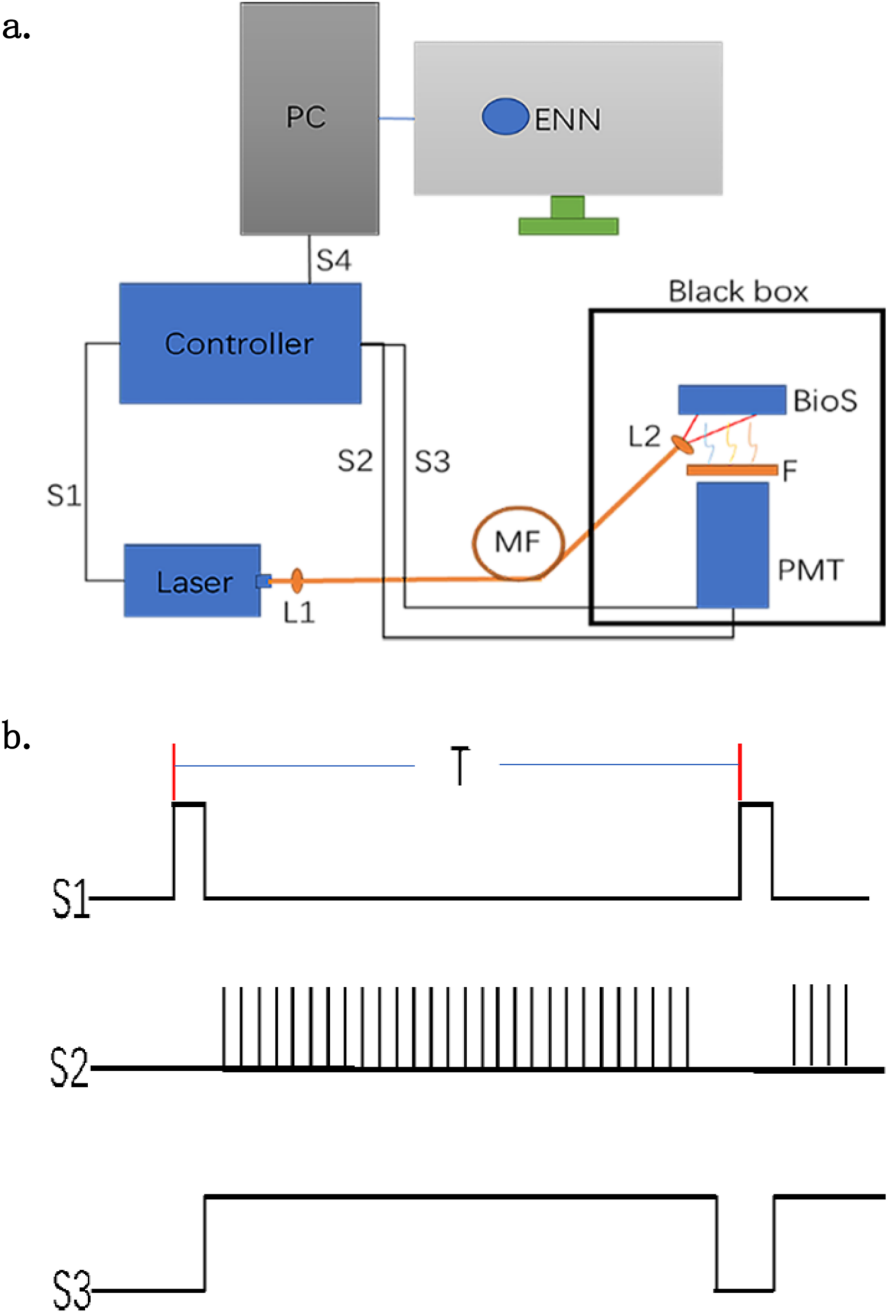
The schematic diagram of delayed luminescence system. *MF* Multimode optical fiber, *PMT* Photomultiplier tube, *BioS* Biological sample, *L1/L2* Lens, *F* Filter, *S1* Laser switching pulse, *S2* PMT photon counting signal, *S3* PMT high voltage control pulse, *S4* USB port.

35The schematic diagram of the system was shown in Fig. 7a. The controller is the center of the acquisition control, and is connected to the PC via a USB port (S4) to transmit commands and data. S1, S2, and S3 are the laser on/off pulses, PMT photon counting signal received, and PMT high voltage control, respectively. The laser is coupled through a multi-mode fiber (MF) through lens (L1) to transmit excitation light (405 nm, 5–30 mW), and the beam size is adjusted by lens (L2) at the exit end. The biological sample (BioS) is illuminated by excitation light pulses and emits DL. The DL emitted by the BioS is received by the photomultiplier tube (PMT) (CR104, Hamamatsu, China) for single photon counting mode, and the spectral sensitivity range of the PMT is from 350 to 650 nm. In the experiment, there is no difference between the DL with too low cell mass and the blank control. Although the curve fit of the DL with high cell mass is very well, the DL value is also very high, which is far beyond the receiving capacity of PMT. To prevent signal oversaturation, a filter (F) is added between the BioS and the PMT, and the F can achieve 50% attenuation. BioS was loaded in a quartz colorimetric cuvette with an optical diameter of 1 mm, and the cuvette was fixed in a black circular holder with a 12 mm diameter hole on the bottom. The pulsed light travelled through the hole to the BioS, and the resulting DL was in turn received by PMT through this hole. In addition, BioS, PMT and other optical devices are placed in a dark box to further prevent external light interference.

The process of data acquisition was periodic. The acquisition cycle (laser pulse period) in the operating parameters was generally chosen to be more than 2 times the delayed luminescence time (time to decay to 30%) for BioS, so that the sample was restored to the initial state before re-excitation. Other parameters to be set included the acquisition delay (D: μs), acquisition time (Tq: μs), number of acquisitions (NT), number of measurements (Nq), etc. Figure 7b presented the working sequence of S1, S2 and S3. A complete picture of just the first cycle was drawn, after which the process was repeated. In one acquisition period (T), the laser emits Nq pulsed beams. The laser was turned on first, and after irradiating the sample through S1 for a certain period of time, the laser was turned off, and high voltage was delivered to the PMT through S3. It takes at least 60 μs to load the high voltage to the PMT to stabilize. Therefore, D is generally greater than 100 μs (For PMTs in counting mode, the excitation beam scatters into the PMT and thus damages the PMT or its circuit, and it is necessary to turn off the PMT with high voltage during the laser emission). After the delay D, the controller starts to count the pulse emitted from PMT. Tq is divided into uniform NT aliquots, and the S2 signal is the clock signal assigned by the internal timer. The clock pulse width is < 5 ns, and the accuracy is adequate. When the timer reaches the set time, S2 is sent and then the count value at this time is saved. After the acquisition completes a cycle, the acquired NT data are sent back to the PC by S4. The data acquired for the Nq times were combined into a DL curve by the ENN software (the conditions for each cycle acquisition were considered to be consistent).

The kinetic curve of DL intensity *I*(t) can be modelled by a hyperbolic function as:

$$I\left( {\text{t}} \right) = \frac{{ I_{0} }}{{\left( {1 + \frac{t}{ \tau }} \right)^{\beta } }}$$

where *I*_0_ is the initial DL intensity, *τ* and *β* are characteristic parameters related to the sample property^37^. The model was used for data acquisition and processing. The experimental data were in good agreement with the DL dynamics described by the formula. The fitting coefficient was more than 0.99.

### Delayed luminescence measurement

For DL measurement, the sample cells were collected by a rapid centrifugation, washed twice with Phosphate-Buffered Saline (PBS) buffer (135 mM NaCl, 4.7 mM KCl, 10 mM Na_2_HPO_4_, 2 mM NaH_2_PO_4_, pH 7.3), and resuspended to 2 × 10^8^ cells/mL. After the cell samples were loaded directly in the quartz colorimetric cuvette, the DL emission was measured immediately. The data acquisition was periodic, and in each period, T was 100 ms, D was 5 ms, Tq was 80 ms, NT was 50, and Nq was 10. The collected DL data was sent back to the PC. After collection, the software displayed the DL decay curve and all the related parameters were calculated.

### Detection of cell growth

OD600 nm was measured with a spectrometer (UV-2800, UNICO, shanghai, China) to detect cell growth. 2 mL of cell suspension was added to a glass colorimetric cuvette with a diameter of 1 cm. The wavelength was set at 600 nm and then measured.

### Detection of cell vitality

The yeast cells were cultured on 96- well plates (Costar, USA) with 8 × 10^5^ cells per well and a final volume of 200 μL. 20 μL Cell Counting Kit-8 (CCK-8; Beyotime, Nantong, China) was added to each well. After 2 h incubation at 30 °C, OD at 450 nm for each sample was measured by using a microplate spectrophotometer reader (Infinite M200, TECAN, Switzerland).

### Detection of mitochondrial morphology

Mito Tracker Red CMXRos (Invitrogen, USA) was used to detect mitochondrial morphology. Yeast cells were washed twice in PBS buffer, then harvested and resuspended in PBS buffer. Mito Tracker Red CMXRos was added to cells at a final concentration of 150 nM and then incubated for 20 min at 30 °C. The cells were washed and suspended in PBS buffer, and applied to a microscopic slide. Place a coverslip on top of the cells and seal the edges of the coverslip with nail polish. The mitochondrial morphology was observed by laser confocal microscope (A1R HD25, Nikon, Japan) (λex = 561 nm, λem = 595 nm), and the image of mitochondrial morphology was analyzed by NIS-Elements Viewer software.

### Detection of mtDNA copy number

1–2 × 10^8^ cells were collected and the total DNA of the cells was extracted using Dr. Gentle ™ (from Yeast) High Recovery Yeast extraction kit (Takara, Japan) according to the manufacturer's instructions. The total DNA concentration was measured using a Nanophotometer (N 60, Implen, Germany) and adjusted to a final concentration of 100 ng/μL. Quantitative Real-Time PCR system (QuantStudio 3, ABI, USA) was used for qPCR. Primers for yeast mitochondrial Mito (forward, 5′-ttg AAG CTG TAC AAC CTA cc-3′; Reverse, 5′-CCT GCG ATT AAG GCA TGA TG-3′) and primers for ACT1 (forward, 5′ -CAC CCT GTT CTT TTG ACT GA-3′; Reverse, 5′-CGT AGA AGG CTG GAA CG TTG-3′) were used. Each reaction system consisted of 1 μL total DNA, 7.4 μL ddH_2_O, 10 μL PCR Mix (PowerUP SYBR Green Master Mix, ABI) and primers with a final concentration of 0.8 μM. The reaction was carried out in a 96-well LightCycler plate (Roche) using the following cycle procedure: 50 °C for 2 min; then 95 °C for 2 min; then 40 cycles of 95 °C for 1 s and 60 °C for 30 s. Each sample was repeated three times. By determining the Ct threshold, the relative mtDNA copy number change was calculated using the comparative ΔΔCt method^38^.

### Detection of ATP

The content of intracelluar ATP was determined by HPLC system (1260 Infinity, Agilent Technologies, Germany). Sample containing 2 × 10^7^ cells in Eppendorf tube was centrifuged for 5 min at 10,000 rpm and 4 °C. The pellet was treated with 360 μL 6% perchloric acid solution at 4 °C for 10 min and then centrifuged at 10,000 rpm and 4 °C for 5 min. 300 μL supernatant was neutralized with 40 uL K_2_CO_3_ (2 mol/L), and the sample was tested after filtration. The solution of 0.1 mol/L KH_2_PO_3_ and methanol (95/5, V/V) was applied as the mobile phase. The detection wavelength was 254 nm. The flow rate was 0.6 mL/min.

### Detection of OCR

The phosphorescent oxygen-sensitive probe, MitoXpress (Cayman, USA), was used to detect OCR. The experimental process was as follows: Reconsititue MitoXpress probe in 1 mL of ultrawater to produce a stock solution with a final concentration of 1 umol/L. Take a 384-well plate (Corning, USA) and place it on a plate heater equilibrated to 30 °C. Add 6 × 10^5^ cells and 1.25 μL MitoXpress probe and SC medium into the well giving a final volume of 40 μL. Then add 100 μL of pre-warmed heavy mineral oil (Cayman, USA) to each well. The fluorescent signal was detected by fluorescent plate reader (FLUOstar-ACU; BMG, Germany).

### Detection of MMP

Rho123 (Beyotime, Nantong, China) was used to detect MMP. Rho123 dye was added to the cell suspension at a final concentration of 2 μg/mL, and the cells were further incubated at 30 °C for 20 min. Then the cells were washed twice with PBS and finally resuspended in PBS. The fluorescence was detected by Flow Cytometry (CytoFLEX, Beckman, China) (FITC channel).

### Statistical analyses

The DL curve was smoothened by using ENN software developed by our company. The *I*_0_ was expressed as mean ± standard deviation, differences between the means were analyzed by using an ANOVA (SPSS version 25; IBM Corp., Armonk, NY) with Tukey’s test, and *p* < 0.05 indicated a statistically significant difference. Group classification according to *I*_0_ was analyzed by k-mean clustering method. The correlation between the *I*_0_ and biochemical parameters was analyzed using Pearson correlation coefficient. Other data were also expressed as mean ± standard deviation of three independent experiments.

## Data Availability

The datasets used and/or analyzed during the current study are available from the corresponding author on reasonable request.

## References

[1] Godlewski M, Zenon Rajfur Z, Sławiński J, Kobayashi M, Inaba H (1993). Spectra of the formaldehyde-induced ultraweak luminescence from yeast cells. J. Photochem. Photobiol. B.

[2] Cifra M, Pospíšil P (2014). Ultra-weak photon emission from biological samples: Definition, mechanisms, properties, detection and applications. J. Photochem. Photobiol. B..

[3] Baran I (2010). Effects of menadione, hydrogen peroxide, and quercetin on apoptosis and delayed luminescence of human leukemia Jurkat T-cells. Cell Biochem. Biophys..

[4] Brizhik L, Musumeci F, Scordino A, Tedesco M, Triglia A (2003). Nonlinear dependence of the delayed luminescence yield on the intensity of irradiation in the framework of a correlated soliton model. Phys. Rev. E. Stat. Nonlin. Soft. Matter. Phys..

[5] Hao OY (2014). The application of ultra-weak photon emission in dermatology. J. Photochem. Photobiol. B..

[6] Harms FA (2011). Oxygen-dependent delayed fluorescence measured in skin after topical application of 5-aminolevulinic acid. J. Biophotonics.

[7] Hua B, Lin L, Ping C, Tang G (2007). Photo-induced delayed luminescence of human serum and its dependence on excitation conditions. Proc. SPIE..

[8] Stolz P, Wohlers J, Mende G (2019). Measuring delayed luminescence by FES to evaluate special quality aspects of food samples—An overview. Open Agric. J..

[9] Mielnik L, Asensio C (2018). Using delayed luminescence to characterize humic acids from lake sediments. J. Soils Sediments.

[10] Scordino A, Triglia A, Musumeci F, Grasso F, Rajfur Z (1996). Influence of the presence of atrazine in water on the in-vivo delayed luminescence of acetabularia acetabulum. J. Photochem. Photobiol. B..

[11] Sung BK, Yi SH, Lee CH, Yang JM, Yang JS (2018). Delayed luminescence of biophotons from plant leaves. J. Opt. Soc. Korea.

[12] Garofalo RT, Moraes TA, Gallep CM (2010). Germination capability of wheat seeds in correlation with delayed luminescence intensity. Tech. Dig. Ser. Opt. Soc. Am..

[13] Vesetova TV, Veselovsky VA, Rubin AB, Bochvarov PZ (2010). Delayed luminescence of air-dry soybean seeds as a measure of their viability. Physiol. Plant..

[14] Sun M (2019). Characterization of ginsenoside extracts by delayed luminescence, high-performance liquid chromatography, and bioactivity tests. Photochem. Photobiol. Sci..

[15] Sun M (2016). Delayed luminescence: An experimental protocol for Chinese herbal medicines. Luminescence.

[16] Lanzano L, Scordino A, Privitera S, Tudisco S, Musumeci F (2007). Spectral analysis of delayed luminescence from human skin as a possible non-invasive diagnostic tool. Eur. Biophys. J..

[17] Joon-Mo Y, Chunho C, Kwang-Sup S (2006). Delayed luminescence characteristics of human hands. Hankook Kwanghak Hoeji.

[18] Scordino A (2014). Ultra-weak delayed luminescence in cancer research: A review of the results by the ARETUSA equipment. J. Photochem. Photobiol. B..

[19] Mik EG (2006). Mitochondrial PO_2_ measured by delayed fluorescence of endogenous protoporphyrin IX. Nat. Methods.

[20] Baran I (2012). Detailed analysis of apoptosis and delayed luminescence of human leukemia Jurkat T cells after proton irradiation and treatments with oxidant agents and flavonoids. Oxid. Med. Cell. Longev..

[21] Kim HB, Baik KY, Choung PH, Chung JH (2017). Pulse frequency dependency of photobiomodulation on the bioenergetic functions of human dental pulp stem cells. Sci. Rep..

[22] Baran I (2013). Mitochondrial respiratory complex I probed by delayed luminescence spectroscopy. J. Biomed. Opt..

[23] Scordino A (2014). Delayed luminescence to monitor programmed cell death induced by berberine on thyroid cancer cells. J. Biomed. Opt..

[24] Kim HW (2005). Spontaneous photon emission and delayed luminescence of two types of human lung cancer tissues: Adenocarcinoma and squamous cell carcinoma. Cancer Lett..

[25] Warburg O (1928). The chemical constitution of resporation ferment. Science.

[26] De Deken RH (1966). The crabtree effect: A regulatory system in yeast. J. Gen. Microbiol..

[27] Olivares-Marin IK, Gonzalez-Hernandez JC, Regalado-Gonzalez C, Madrigal-Perez LA (2018). *Saccharomyces cerevisiae* exponential growth kinetics in batch culture to analyze respiratory and fermentative metabolism. J. Vis. Exp.

[28] Michaela C (2014). Nutrient sensing and signaling in the yeast *Saccharomyces cerevisiae*. FEMS Microbiol. Rev..

[29] Zhang T, Bu P, Zeng J, Vancura A (2017). Increased heme synthesis in yeast induces a metabolic switch from fermentation to respiration even under conditions of glucose repression. J. Biol. Chem..

[30] Zcan S, Dover J, Johnston M (1998). Glucose sensing and signaling by two glucose receptors in the yeast *Saccharomyces cerevisiae*. EMBO J..

[31] Koštejnová L (2021). Cultivation of *Saccharomyces cerevisiae* with feedback regulation of glucose concentration controlled by optical fiber glucose. Sensor.

[32] Francesca G (1804). Effect of different glucose concentrations on proteome of *Saccharomyces cerevisiae*. Biochim. Biophys. Acta.

[33] Pham H, Larsson G, Enfors SO (1998). Growth and energy metabolism in aerobic fed-batch cultures of *Saccharomyces Cerevisiae*. Biotechnol. Bioeng..

[34] Bagamery LE, Justman QA, Garner EC, Murray AWA (2020). Putative bet-hedging strategy buffers budding yeast against environmental instability. Curr. Biol..

[35] Joliot P, Joliot A (1980). Dependence of delayed luminescence upon adenosine triphosphatase activity in *Chlorella*. Plant Physiol..

[36] Grasso R (2016). The delayed luminescence spectroscopy as tool to investigate the cytotoxic effect on human cancer cells of drug-loaded nanostructured lipid carrier. Biophotonics Photonic Solutions Better Health Care.

[37] Gu Q (2016). Biophotonics.

[38] Livak KJ, Schmittgen HD (2001). Analysis of relative gene expression data using real-time quantitative PCR and the 2^−^^ΔΔCT^ Method. Methods.

